# Homoepitaxy Growth of Single Crystal Diamond under 300 torr Pressure in the MPCVD System

**DOI:** 10.3390/ma12233953

**Published:** 2019-11-28

**Authors:** Xiwei Wang, Peng Duan, Zhenzhong Cao, Changjiang Liu, Dufu Wang, Yan Peng, Xiaobo Hu

**Affiliations:** 1 State Key Laboratory of Crystal Materials, Shandong University, Jinan 250100, China; xwang21@126.com (X.W.); 13256998266@163.com (P.D.); wangdufu@163.com (D.W.); 2 Jinan Diamond Technology Co. Ltd, Jinan 250101, China; 13608927651@163.com (Z.C.); owlchj@163.com (C.L.)

**Keywords:** single crystal diamond, Homoepitaxy growth, 300 torr

## Abstract

The high-quality single crystal diamond (SCD) grown in the Microwave Plasma Chemical Vapor Deposition (MPCVD) system was studied. The CVD deposition reaction occurred in a 300 torr high pressure environment on a (100) plane High Pressure High Temperature (HPHT) diamond type II *a* substrate. The relationships among the chamber pressure, substrate surface temperature, and system microwave power were investigated. The surface morphology evolution with a series of different concentrations of the gas mixture was observed. It was found that a single lateral crystal growth occurred on the substrate edge and a systemic step flow rotation from the [100] to the [110] orientation was exhibited on the surface. The Raman spectroscopy and High Resolution X-Ray Diffractometry (HRXRD) prove that the homoepitaxy part from the original HPHT substrate shows a higher quality than the lateral growth region. A crystal lattice visual structural analysis was applied to describe the step flow rotation that originated from the temperature driven concentration difference of the C_2_H_2_ ion charged particles on the SCD center and edge.

## 1. Introduction

Diamond is gaining more attention for its outstanding optical, electrical, mechanical, and thermal properties. Since the first successful synthesis of diamond by General Electric more than half a century ago, diamond has attracted researchers to develop new growth technologies and extend multiple applications. Currently, High Pressure High Temperature (HPHT) and Microwave Plasma Chemical Vapor Deposition (MPCVD) technologies are widely used to manufacture large, single crystal diamonds with good quality and clarity. High-quality single crystal diamond (SCD) is available in superhard cutting tools, optical components, semiconductor and high-power electronics, and even in quantum applications [1,2,3,4].

Although the HPHT method still produces the overwhelming majority of a single crystal diamond, the metallic impurity incorporation and high dislocation density in crystal edges limits its further application in the semiconductor and quantum field. MPCVD technology has become a mature and reliable method for high quality SCD growth since it has the advantages of the control of the nitrogen concentration and dislocation density [5]. Many studies have indicated that the surface morphology, growth rate, and crystal quality depend on the internal reaction chamber structural parameters such as the substrate holder, input gas mixture, and chamber pressure. Accordingly, the growth mechanism of high quality SCD was proposed. Qi Lang et al. reported a growth rate of 165 μm/h by doped nitrogen into the reaction chamber [6]. Nad et al. compared different sizes of pocket holders to control the substrate surface morphology under a chamber pressure of 240 torr. Silva et al. showed that increasing the process pressure can increase the diamond growth rate and improve the crystal quality [7,8]. F. Lloret studied the stratigraphy of SCD lateral growth on a three-dimensional structure and designed a geometric model for the growth sector configuration [9,10,11]. Among these, the reaction pressure plays an important role in the overall carbon deposition and homoepitaxy growth because it determines the shape, distribution, and available area of the activated plasma. Due to the limitation of the CVD system parameter, achieving high chamber pressure during the growth meets a particular set of problems and uncertainties [12]. Most research studies have reported that pressure was controlled in a range of 100–250 torr [13,14,15] during diamond crystal growth. Growth pressures higher than 250 torr are still rarely seen.

In this study, we focused on SCD growth under a reaction pressure of 300 torr. The surface morphology and growth rate were studied in order to optimize the growth rate to ensure the crystal quality.

## 2. Materials and Methods

### 2.1. Preparation of the CVD Diamond Substrate

High quality HPHT diamond crystal, which is 7 × 7 mm in size, was grown in a cubic press system by Jinan Diamond Technology Co., Ltd. The origin diamond was type I *b* with a nitrogen concentration of about 10^19^/cm^3^, as measured by Secondary Ion Mass Spectroscopy (SIMS) from EAG laboratories (Shanghai China) [16]. After the HPHT growth, the diamond crystal was boiled in the mixed solution of perchloric acid, sulfuric acid, and hydrochloric acid for 3 h to remove the dirt covered on the crystal surface (All the above acids are from Aladdin, Shanghai, China). The HPHT diamond substrate plate was cut parallel to the (100) surface by the laser sawing system. The substrate was 5.97 mm × 5.57 mm in size, and the thickness was 0.61 mm. Mechanical polishing was applied to remove the surface cracks generated during the sawing process. Ultrasonic cleaning of the methanol and acetone separately for half an hour was used to remove the organic substances on the surface of the substrate.

### 2.2. The Etching of the CVD Single Crystal Substrate

To achieve a nitrogen-free growth environment during the entire process, the chamber was evacuated to 0.1 torr by dry pump before the supply of the hydrogen. Etching was followed for half an hour under 300 torr pressure and 900 °C in a pure hydrogen environment to remove the impurities and defects caused by the mechanical polishing.

### 2.3. Growth Parameter Investigation

After the etching, the CVD diamonds were grown on the substrates with different parameters including the temperature, pressure, and methane concentration. The relationship between the temperature, pressure, and microwave power was studied systematically to understand the system’s plasma energy distribution and absorbance. All diamond substrates were laser cut to 0.5 mm thickness and grown in the reaction chamber with a methane concentration of 3%.

The surface morphology and growth rate were investigated at different methane concentrations under a pressure of 300 torr and 1150 °C with hydrogen flow at 600 sccm for 4 h of growth. The methane concentration was varied from 2% to 5% in order to get an acceptable surface morphology and growth rate to optimize the SCD growth.

### 2.4. The Growth of the CVD Diamond Layer

After that, the SCD layer was grown for 4 h after hydrogen etching of the substrate at 900 °C for half an hour. The chamber pressure was 300 torr, while the substrate temperature was kept at 1150 °C with a gas mixture of CH_4_:H_2_ to 18:600 sccm. During the CVD reaction, the thickness of the sample increased and approached the lower edge of the plasma. The substrate surface temperature increased, which changed the thermodynamics balance for the carbon deposition and hydrogen etching. A thermo pyrometer was used to measure the in situ substrate temperature. Since the sample thickness increases during the CVD reaction, the self-control recipe mode was activated during the growth period in order to self-adjust the input microwave power to maintain the substrate surface temperature through the feedback of the thermo pyrometer. The temperature variation range was less than 10 °C throughout the whole period of growth.

### 2.5. The Surface Morphology and Crystal Quality Analysis

A confocal laser scanning microscope OLS-4000 from Olympus (Tokyo, Japan) was employed to examine the top surface morphology of the diamond. Single crystal quality was assessed via HRXRD by D8 Discover operating with CuKα_1_ radiation with an anode on a fixed power supply at 40 kV/40 mA from Bruker. The room temperature Raman spectra were obtained by the LabRAM HR800 system of Horiba Jobin Yvon (Paris, France) with a 514 nm solid laser as the excitation source to determine the phase structure.

## 3. Results and Discussion

### 3.1. CVD System for SCD Growth

**Figure 1:**
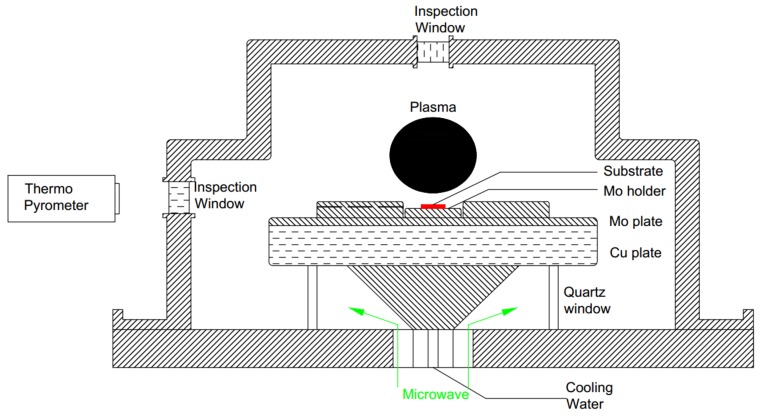
Schematic diagram of the Ardis 300 reaction chamber. The red rectangle on the Mo holder represents the High pressure High Temperature (HPHT) diamond substrate, and the green arrow stands for the direction and for the microwave transmission path.

The System type Ardis 300 is equipped with a 2.45 GHz/6 kW microwave reactor by Optosystems Ltd. The microwaves enter the reaction chamber through the quartz window from the corn shape guide beneath the sample stage. Figure 1 shows a schematic diagram of the structure of the Ardis 300 reaction chamber. The sample stage is made of copper with water channels inside to allow cooling during the reaction. A molybdenum plate is placed on the top of the stage and a specially designed closed type sample holder is located in the center for the substrates [8,17]. Quartz windows are implanted into the stainless steel chamber shell on the top and side wall for inspection. A double interference infrared radiation thermo pyrometer is installed to measure the temperature of the substrate with an emissivity of 0.1 through a slit of 2 mm. In order to get a real-time substrate temperature measurement during the deposition reaction, the system was managed to carve a guide channel hole throughout the encircled ring, which is parallel to the ring surface. The pressure of the reactor was able to reach a maximum of 300.0 torr with an accuracy of 0.1 torr.

### 3.2. The Influence of the Chamber Pressure on the Microwave Power and Substrate Temperature

**Figure 2:**
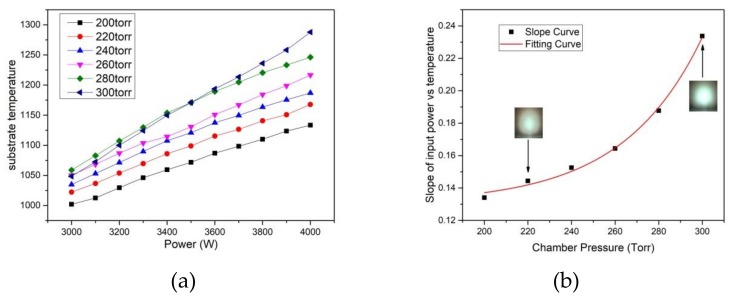
(**a**) Relationship of the microwave power to the substrate temperature during the CVD growth under different high chamber pressures. The chamber pressure varied from 200 to 300 torr. (**b**) Calculated slopes of the curve from Figure 2a at different chamber pressures with fitting. Two photos of the plasma were captured throughout the inspection window under 3800 kW, 1150 °C, 220 Torr, and 300 Torr, separately.

The chamber pressure plays a significant role in determining the growth rate, morphology, and crystal quality prominently in terms of the homoepitaxy and lateral growth during the CVD reaction. In order to obtain optimized parameters for homoepitaxy growth, a stable thermal and electromagnetic field should be guaranteed by considering a series of deposition parameters such as microwave power, chamber pressure, and methane concentration. Figure 2a shows the relationship between microwave power and substrate temperature under different chamber pressures from 200 to 300 torr for Ardis 300. The results indicate that, under the same pressure of the reaction chamber, the substrate temperature increases linearly with the microwave power. The slopes under the different chamber pressures are calculated and fitted in Figure 2b. The slope of the curve increases from 0.134 (200 torr) to 0.234 (300 torr). The fitting curves follow the exponential function calculated as:

(1) y=0.13+6.2×103ex−197.8736.64.

The entire CVD deposition reaction system is a strong self-adaption multi-field environment with different plasma particles generated by the microwave discharge in the chamber. The movement of all deposition-related ionized particles is violently affected by the constantly changing compound electromagnetic field provided by the reactor, which leads to different transmissions of the particles throughout the plasma generation to the deposition position on the diamond substrate. As the chamber pressure increases, the effective availability of the microwave increases to promote the methane and hydrogen to discharge, which causes a dynamic acceleration for both the carbon deposition and etching because of the different partial pressures [18]. The surface temperature difference of the substrate acts as the driving force for the material transformation in the thermodynamic routine. The present experimental result indicates a significant increase in the magnitude of the slope of microwave power over the substrate temperature since the reaction pressure increases. It reveals that there is enormous potential for plasma discharge absorption for the supplied power in an even higher pressure environment (over 200 torr).

The plasma photos in Figure 2b were captured under the same microwave power of 3800 W and temperature of 1150 °C. It shows that the plasma diminished in size and brightened in color from 220 to 300 torr separately. In this procedure, the plasma energy density increases. As a consequence, the charged concentration of the carbon-atom-related growth species such as CH_3_ and C_2_H_2_ increases. No clear point microwave discharge around the substrate and Molybdenum ring edge was found during the pressure change in the deposition reaction. The temperature gradient will be changed accordingly and will bring a different thermodynamic approach to the substrate homoepitaxy and the edge lateral overgrowth [19].

### 3.3. The Influence of the Methane Concentration on the Morphology and Growth Rate

**Figure 3:**
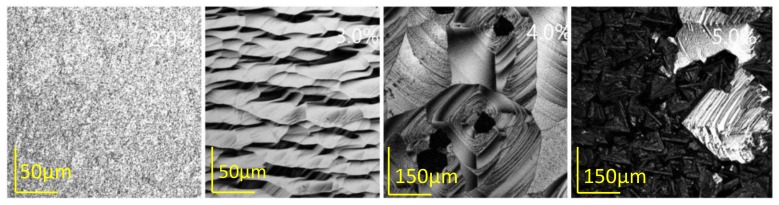
Surface morphology evolution of the substrates grown under the environment of CH_4_:H_2_ at 2.0%, 3.0%, 4.0%, and 5.0%. The substrates were grown at 1150 °C and 300 torr. The images were captured by the confocal laser scanning mode of the confocal laser scanning microscope.

**Figure 4:**
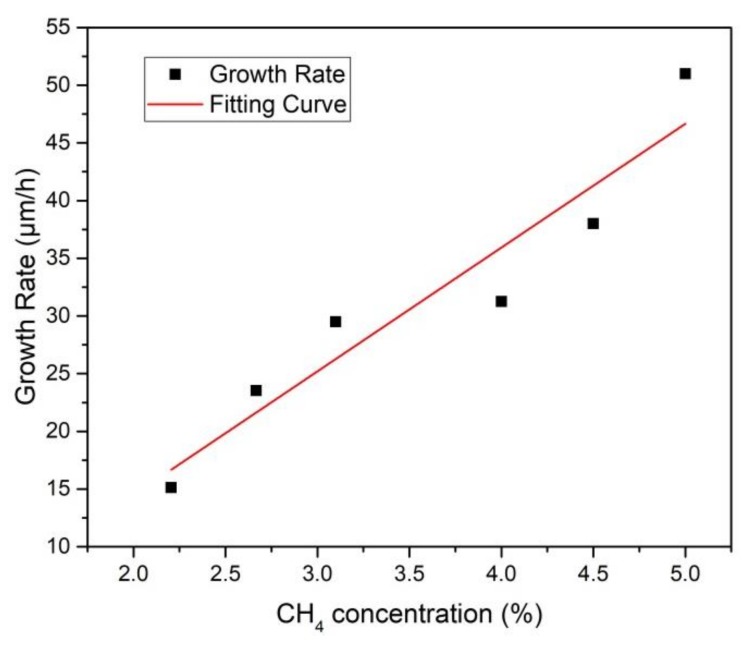
Relationship between the methane concentrations versus the deposition growth rate. The growth rate was calculated by the average measurement of the four corners and center after the growth and divided by the time. The red line is the fitting curve of the dot distribution.

As the only carbon atom provider, the methane concentration is particularly emphasized for the SCD growth with the CVD method. The optimal methane concentration leads to a polycrystalline-free layer surface with high quality and an acceptable growth rate. Besides the growth rate, another significant parameter, known as the edge boundary of the substrates, should be fully considered, which requires a controllable, sequential, and well organized epitaxial lateral overgrowth to exclude the growth of the polycrystalline. Several typical surface morphologies are exhibited in Figure 3 for methane concentrations from 2.0% to 5.0%. Harris and Goodwin [17,20] described the CVD diamond growth reaction using a simple model in which the growth rate depended on a metastable competition between the hydrogen etching and the methyl radical deposition. Figure 4 shows the relationship between the methane concentration and the growth rate, which were obtained at a pressure of 300 torr and 1150 °C and a hydrogen flow of 600 sccm throughout the entire experiment. An almost linear relationship for the methane concentration with the growth rate was present. When the methane concentration was 2%, which shows a recessive role in the competition, the substrate exhibited a multi-hole structure with plenty of etch pits on the diamond surface. The violent etching reaction restrained the appearance of the homoepitaxy step growth, which corresponded to a low growth rate of about 15.6 μm/h. When the methane concentration increased to 3%, the surface showed parallel and regular growth steps flow along the [100] direction, which indicates a well-controlled equilibrium of the carbon transportation to the substrate from the charge plasma and, meanwhile, indicates an organized arrangement of carbon atoms. As the methane concentration increased gradually, polycrystalline appeared and the different domains were present on the surface.

The surface was severely covered with the disordered polycrystalline scale clusters when the methane concentration increased to 5%, which proves that over-dosed methane led no approach to a compromised diamond quality, even though this sample achieved a high growth rate of 51 μm/h.

In order to obtain a higher quality homoepitaxy of a single crystal diamond layer with a reasonable growth rate, we decided to limit the methane concentration to 3% during the deposition growth.

### 3.4. The Homoepitaxy Growth of the Single Crystal Diamond 

After determining the temperature, pressure, and the methane concentration, we tried to optimize the reaction parameter to achieve a high-quality CVD single crystal homoepitaxy diamond layer. The average growth rate was 27.12 μm/h, which almost matches the result we obtained from Figure 4.

**Figure 5:**
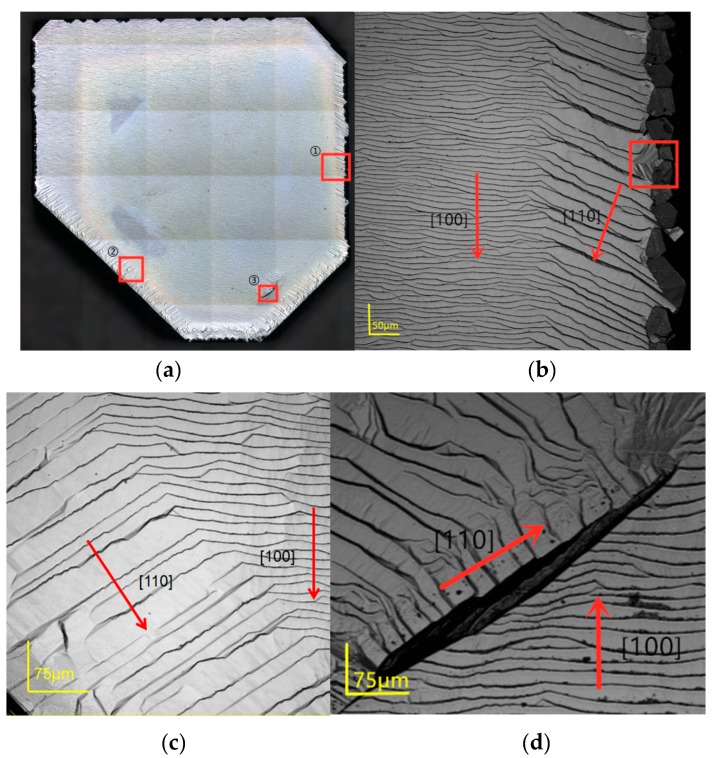
(**a**) Image of the entire diamond substrate surface after 4 h of growth at 300 torr/1150 °C, CH_4_:H_2_ = 16:600 sccm. Three detailed images are labeled as positions 1, 2, and 3. (**b**) The image of the sample of 50× amplification at position 1. The red box reveals the pyramid structure in the edge neighboring the polycrystalline. (**c**) The image of the 50× amplification at position 2. (**d**) The image of the 50× amplification at position 3 with a deep crack in the center. The red arrows show the direction of the step flow.

Figure 5a is an image of the diamond surface taken by the confocal laser scanning microscope. Most of the surface area is covered with a regular step flow. Several large dark inclusions can be observed in the center of the sample, which represents the metal compound catalysts left in the crystal during the HPHT growth. The light yellow boundary shows the original HPHT diamond substrate edge beneath the as-grown sample. Lateral growth can be clearly observed by comparing the edge of the sample with the HPHT substrate boundary. Figure 5b–d show the photos captured in the laser scan mode. Two domains can be observed from Figure 5b,c in different positions of the sample. The center domain steps are along the [100] orientation, which can be easily observed on the surface of SCD homoepitaxy layers. The step width is about 10–16 μm. However, the steps on the edge domains go along the [110] orientation with a width of around 18–26 μm. Clear step merging along the [110] orientation can be observed on the edge lateral growth. After comparing the diameter of the as-grown sample with the original substrate before and after the growth, we confirmed that the [100] orientation domain on the edge is generated by the lateral growth. The right edges in Figure 5b show the generation of the polycrystalline. The polycrystalline can become an induction of the un-epitaxial features on the edges and corners and leads to the formation of asymmetric nuclei, which exhibit the pyramid structure revealed in the red block [21,22]. Shreya Nad et al. reported that the non-uniform surface temperature shall be generated at the sharp edges of the substrate during the CVD growth. This phenomenon may be caused by the difference in the plasma density in the corresponding ambient region between the substrate, holder surface, and the local heat transmission to the cooling water, which leads to the generation of higher index symmetric planes such as (110) and (111) in pyramid structures or polycrystalline [7].

Figure 5d shows the crack along the [100] direction, which may be caused by the metal inclusion. A. Ababou et al. reported that metal inclusions play a role in the CVD homoepitaxy growth by accelerating the formation of the sp^2^ phases because of non-epitaxial growth. The dislocations brought by the metal inclusions will be propagated from the center of the substrate to the epitaxy layer throughout the entire growth process even if they are concealed inside. The stress will be relieved as the defects accumulate to generate enough lattice mismatch before breaking and cracking [23].

### 3.5. CVD Homoepitaxy Growth Diamond Quality

**Figure 6:**
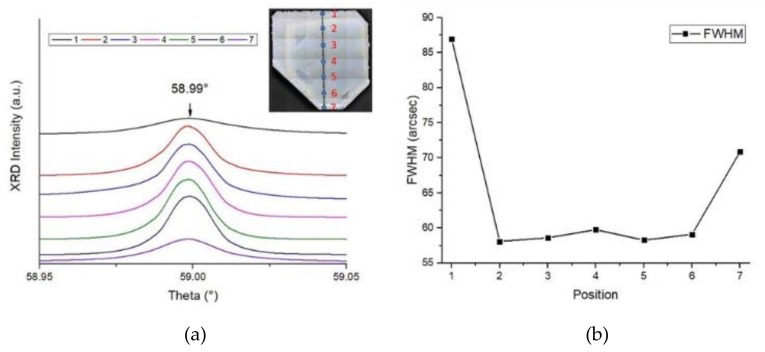
(**a**) HRXRD rocking curve of the (400) direction symmetric reflections of the location points from 1 to 7. The distance of each neighbor point was designed to be 1 mm. As a result, points 1 and 7 were located on the lateral growth area of the CVD layer. (**b**) The XRD FWHM of the location positions from points 1 to 7.

**Figure 7:**
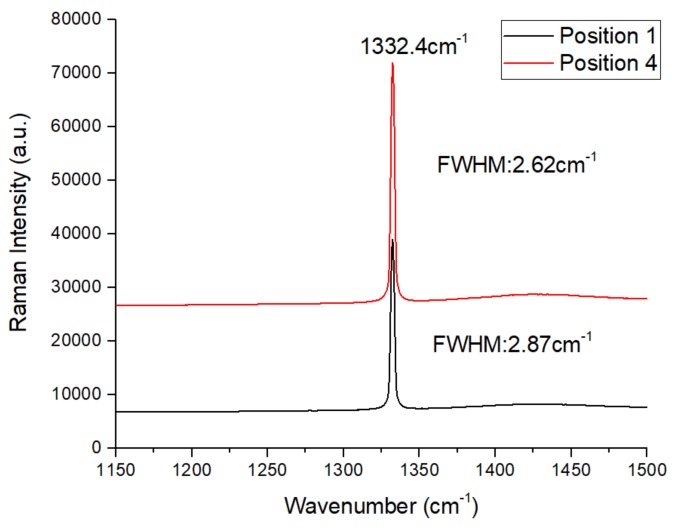
Raman spectrum of the CVD homoepitaxy layer at room temperature, the black and red lines correspond to location points 1 and 4, respectively.

In order to understand the quality of the homoepitaxy layer and the lateral edge, we measured the rocking curves of the High Resolution X-Ray Diffractometry (HRXRD) and Raman spectra of the samples. Figure 6a shows the X-Ray Diffractometry (XRD) rocking curves of (400) reflection on different sections of the CVD substrate. The section positions are labeled from 1 to 7 corresponding to those points on the substrate. The distance between two measured points is 1 mm. Since the distance of the SCD layer in the labeled direction was about 6.13 mm, points 1 and 7 were on the lateral edges, and points 2 to 6 were on the homoepitaxy layer from the original substrate, as designed. The FWHM values of the rocking curve of points from 1 to 7 are shown in Figure 6b. The layer structural quality is similar to that of points 2 to 6. The FWHM values of the rocking curve for homoepitaxy layer were about 57–59″ while those of the edge section were 86.98″ and 70.98″ at points 1 and 7, respectively. The measurement was accomplished in a high-intensity configuration of the variable slit mode and indicates a high uniformity on the homoepitaxy growth and an imperfect crystalline quality on the lateral edges. Figure 7 shows the Raman spectra of the CVD layer at positions 1 and 4, which represents the structural quality of the homoepitaxy and lateral sections. Two spectra are dominated by a single sharp peak at 1332.43 cm^−1^, which reveals that the internal stress did not increase by an order of magnitude. No peak related to the sp^2^ carbon phase can be observed. The full width at the half maximum (FWHM) value of point 1 is somewhat larger than that of position 4, which indicates higher residual stress on the lateral edges than in the center. This matches the XRD rocking curve result. This means that the stress around position 4 is smaller than that of point 1. Both of the XRD rocking curves and Raman spectra prove that the sample layer shows a high crystal quality in both the center and edge parts of the sample, while the homoepitaxy region inherited from the HPHT substrate presents a high crystal quality with fewer defects and low internal stress compared with the edge overgrowth.

### 3.6. Crystal Lattice Visual Structure Analysis of the Step Flow Rotation

**Figure 8:**
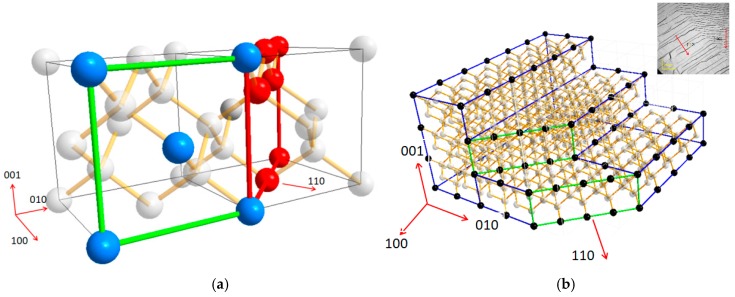
(**a**) Atom lattice visual structure of the diamond double unit molecule cell. The green lines are links of the carbon molecules on the (100) surface. The red lines are the (110) surface. (**b**) The lattice visual structure indicates the edge rotation transformation from the direction [100] to [110]. The black atoms are the carbon atoms on the surface of the substrate. The blue lines contact the atoms on the (100), (010), and (001) surfaces. The green lines link the carbon atoms and form the faces vertical to the [110] direction.

In order to understand the growth mechanism of the homoepitaxy and lateral growth as well as the step flow rotation on the edges, diamond lattice structure analysis was used to describe what happened on the edges. Figure 8a shows the basic double unit cells of the carbon atom’s cubic region at the center of the face during diamond phase formation. The blue atoms form the (100) unit face while the red ones are in (110) faces. After the deposition of the (100) surface, following the original step flow, the step flow rotates 45° in the [110] direction on the edge of the sample. Figure 8b simulates the step flow of the CVD sample edge to describe the appearance of the step rotation. The black atoms are on the sample surface linked by the blue lines, forming the [100] orientation step surface. The green surface represents the (110) surface. Alexandre Tallaire et al. reported this phenomenon generated from the (100) face to the (110) face on the lateral side, which is almost the same as our result of the transformation of the step flow. Their team explained that the face rotation should evolve due to the temperature difference between the center and the edge [13,24]. E.V. Bushuev et al. also considered the dependence of the diamond epitaxial growth rate on the substrate temperature. They predicted that the microwave power would play a minor role in the growth kinetics via the plasma distribution [25]. C.C. Battaile et al. reported the CH_3_ and C_2_H_2_ groups are the main source for the growth of the (100) and (110) surfaces, respectively, during the CVD diamond deposition. Furthermore, acetylene is particularly important for aiding in the formation of multi-carbon clusters on the nuclei on the different orientation surfaces [26,27]. In sum, we predict that the step flow rotation of our sample was caused by the anomalous distribution of the C_2_H_2_ on the substrate surface. Different from the CH_3_ guided nuclei, which dominated the primary surface for the crystal growth, for the C_2_H_2_ guiding mode, two carbon atoms were first combined to form a double bond bridge onto the ravine of the (110) surface before further deposition, which resulted in a 45° guide to the lattice formation. As a result, the generation of the C_2_H_2_ cluster needed extra time and energy to generate equivalent growth on the (110) surface. The thermodynamic of the growth of the center and edge of the sample were driven by the temperature gradient provided by the optimized microwave plasma. The higher temperature of the edge provided the required surface energy for the growth of the (110) surface. According to the Gibbs–Wulff principle, the (110) plane has a lower surface free energy than that of the (100) surface. Meanwhile, it has a lower growth rate as well [24]. As the time elapsed during the reaction, the (110) surface was found to have a lower surface energy level than that of the (100) surface on the edge and lateral growth regions. Additionally, the lower growth rate of the (110) surface led to the step merging at the rotation site from the [100] to [111] steps. Similar morphology can be observed in Figure 5d because of the poor thermo conductivity of the surface crack. The temperature on the crack edge was higher than that of the other regions. It may have also been caused by the plasma point charge.

## 4. Conclusions

In this paper, we introduced an HPHT diamond substrate as the seed of the homoepitaxy growth in a CVD system under a high chamber pressure of 300 torr. The relationships among the input power, substrate temperature, and chamber pressure were first studied in order to understand the plasma behavior during the deposition reaction. A series of methane concentrations, growth rates, and surface morphologies were investigated to optimize the growth parameter. After that, a high quality SCD layer was achieved with a 27.12 μm/h growth rate under a 300 torr high pressure environment. The sample surface presented a regular uniform [100] orientation surface step flow in the original substrate area and a [110] orientation step in the lateral growth edges. Raman spectroscopy and the HRXRD rocking curve were used to investigate the internal stresses and crystal quality of both the homoepitaxy and lateral layers. The lattice visual structure model was employed to explain the step flow rotation in the lateral growth edge. Different from the CH_3_ guided [100] orientation deposition model, C_2_H_2_ acted as the preponderant growth source of the [100] orientation step flow, which relies on higher energy provided by the higher temperature on the substrate edges.
